# Comparison of Children’s Preferences for Dental Injectors and Their Impact on Cooperation During Local Anesthesia Injection: An Observational Study

**DOI:** 10.7759/cureus.86889

**Published:** 2025-06-27

**Authors:** Irsam Haider, Malik Adeel Anwar, Hira Zaman, Maryam Virda, Aneeqa Ali, Farheen Komal, Zain ul Abidin, Imza Batool

**Affiliations:** 1 Department of Operative and Pediatric Dentistry, University College of Dentistry, The University of Lahore, Lahore, PAK; 2 Department of Biomedical Engineering, Binghamton University (State University of New York), Binghamton, USA; 3 Department of Oral Pathology and Oral Diagnostics, University College of Medicine and Dentistry, The University of Lahore, Lahore, PAK; 4 Department of Operative and Pediatric Dentistry, University College of Medicine and Dentistry, The University of Lahore, Lahore, PAK; 5 Department of Operative Dentistry, FMH (Fatima Memorial Hospital) College of Medicine and Dentistry, Lahore, PAK; 6 Department of Operative Dentistry, Isra Dental College, Hyderabad, PAK

**Keywords:** behavior rating scale, dental anxiety, distraction techniques, local dental anaesthesia, local dental anesthesia, patient cooperation, pediatric dentistry

## Abstract

Background

Dental fear and anxiety (DFA) are a common challenge in pediatric dentistry, often triggered by fear of pain, unfamiliar instruments, or appearance of dental instruments and injections. This fear significantly affects children’s cooperation during procedures, making local anesthesia (LA) particularly anxiety-inducing.

Objective

This study aimed to evaluate children's level of cooperation during dental procedures in relation to their preference for different types of dental injectors.

Materials and methods

This study involved 150 children randomly assigned into two equal groups using a lottery method. Group A was given a choice between a metal injector and a plastic injector without stickers on it, while Group B chose a metal injector or a plastic injector with stickers around it. Children's preferences were recorded, and their behavior was assessed using the Frankl behavior rating scale during LA administration.

Results

In Group A, 43 (57.3%) children chose plastic injectors without stickers compared to 32 (42.7%) preferring the metal injector. In Group B, 62 (82.7%) children preferred plastic injectors with stickers, whereas only 13 (17.3%) chose metal injectors. Behavioral assessments revealed that children in Group A who preferred the plastic injector without stickers exhibited more positive behavior (n = 29, 67.5%), whereas those who preferred the metal injector showed more negative behavior (n = 10, 31.3%). In Group B, children who preferred plastic injectors with stickers showed more positive behavior (n = 43, 69.3%) when compared with those who chose metal injectors in the same group. Across all age groups, children showed more positive behavior toward plastic injectors, particularly those with stickers on them. This difference was statistically significant in the 4-6 and 7-10 years groups (p = 0.039 and p = 0.011, respectively).

Conclusion

The addition of stickers to plastic injectors significantly improved children's comfort and behavior during dental procedures, particularly in younger children. Older children preferred injectors without stickers, suggesting that age-specific designs enhance cooperation levels and reduce dental anxiety.

## Introduction

Dental anxiety can be defined as a state of apprehension that something dreadful is going to happen in relation to dental treatment [1]. Dental phobia is ranked the fourth most common fear overall [2]. Children who visit dental facilities for treatment frequently exhibit fear and anxiety, a phenomenon known as dental fear and anxiety (DFA). DFA in pediatric patients is often rooted in the fear of the unknown, pain, dental instruments or the dentist themselves [3]. If not effectively managed, DFA can escalate into dental phobia, one of the most prevalent fears, leading to avoidance of the necessary dental care [4]. In fact, research assessing the dental fear among children revealed that 41% of participants exhibited high fear scores, particularly regarding “injections,” “dentist drilling,” and “choking” [5]. Moreover, fear of needles, also known as trypanophobia, is a prevalent concern among pediatric patients, exacerbating anxiety and negatively hampering cooperation during dental procedures [6].

Since its discovery, local anesthesia (LA) has been an integral component of dental treatments, ensuring pain-free procedures and promoting a positive dental experience [7]. However, the sight of dental injectors often triggers fear in children. This fear is largely attributed to their intimidating appearances. Studies suggest that disguising dental injectors, such as by covering them with cartoon-themed stickers, can make them appear less frightening for the child, positively impacting the child’s behavior [8]. Furthermore, providing children with the opportunities to make choices or actively participate in their dental treatment can empower them and reduce anxiety levels, fostering a sense of control, leading to cooperative behaviors [9].

Numerous studies have investigated various methods to reduce dental anxiety in children by altering the appearance of dental injectors by camouflaging them or using plastic alternatives to reduce dental anxiety in children [8,10,11]. In addition to these approaches, incorporating simple behavior management techniques such as tell-show-do, modeling and positive reinforcement, alongside using calming colors and other environmental elements in the clinics can produce positive feelings, reducing the child’s anxiety during their dental treatment, thus increasing their cooperation. These strategies work together to create a more welcoming and less intimidating experience for the child [12-14].

Dentists frequently encounter a wide spectrum of behaviors in children, from highly cooperative to anxious or challenging. Many experts have proposed various scales to classify and assess child behavior in dental settings, such as Venham’s picture scale; face, legs, activity, cry, consolability (FLACC) behavior pain scale; facial image scale (FIS); and visual analog scale (VAS) [8,10,11,15]. Among these is the Frankl behavior rating scale, a four-point scale modified by Wright in 1975 [16]. It is relatively simple and easy to use, as it provides behavior ratings in numerical figures that can be used to scale behavior, and it is widely regarded as one of the most effective tools for assessing children's behavior in dental settings [17].

The purpose of this study was to compare children's preference for dental injectors and assess how these preferences impact their level of cooperation. It also investigated whether specific cues, such as placing friendly stickers on dental injectors, can enhance a child's willingness to cooperate during dental treatments.

## Materials and methods

An observational study was conducted in the department of pedodontics at the University College of Dentistry, the University of Lahore. The study received approval from the Ethical Review Committee under reference no. UCD/ERCA/590. The study was conducted from October 2023 to May 2024. Before participation, informed parental consent was obtained, with consent forms available in both English and Urdu. A sample size of 150 children (75 in each group) was calculated with a 6.5% level of significance and 80% power of test, and by taking the expected percentage of positive attitude toward anesthesia acceptance rating with plastic injector without stickers and with stickers as 20% and 40%, respectively. The sample size was calculated to be 150 [18].

Selection criteria included children of 4 to 12 years of age attending the hospital for treatment involving local infiltration anesthesia. Children who had any systemic condition, were with special healthcare needs, or were unwilling to participate were excluded from the study.

Participants were randomly allocated into two groups using a lottery system. A box containing 75 slips for each group was prepared. Each child was asked to randomly draw a slip from the box, and they were then assigned to the group indicated on their hand-drawn slip.

**Figure 1:**
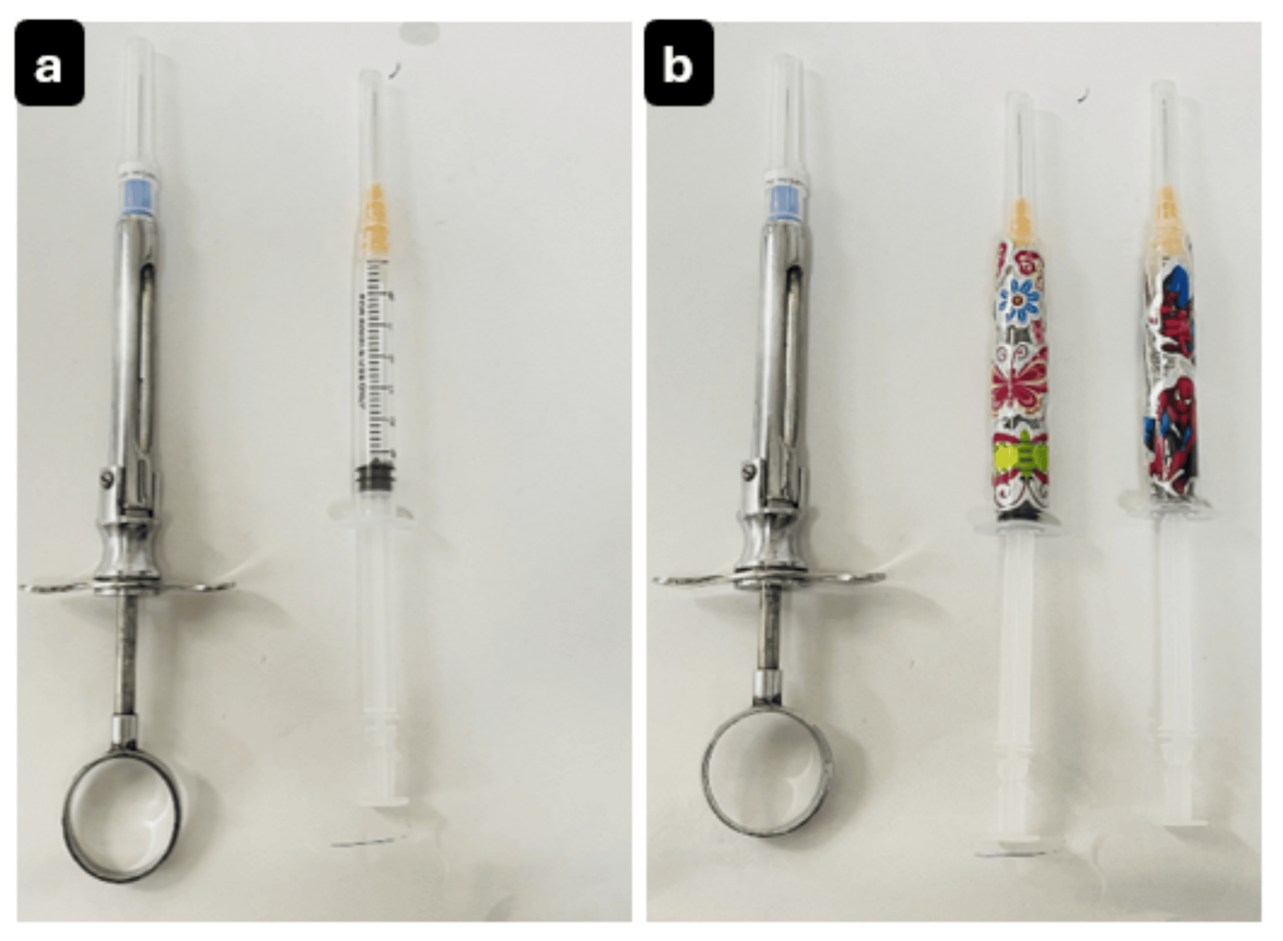
Injectors used in Group A (a) and Group B (b)

Group A consisted of children who were presented with a traditional metal injector and a plastic injector without stickers. Group B included children who were provided with a traditional metal injector and a plastic injector decorated with stickers featuring either a floral (for girls) or superhero (for boys) themed character (Figure 1). All these injectors were equipped with a 27-gauge needle, and lidocaine HCl 2% with adrenaline 1:80,000 (Lignospan special, Septodont, Saint-Maur-des-Fossés, France) was used in both injectors as a local anesthetic.

Once the children were assigned a group, they were presented with injectors corresponding to their allocated group and allowed to freely choose one without any influence from the dentist or their parents. They were then asked to explain the reasons for choosing a certain injector.

**Figure 2:**
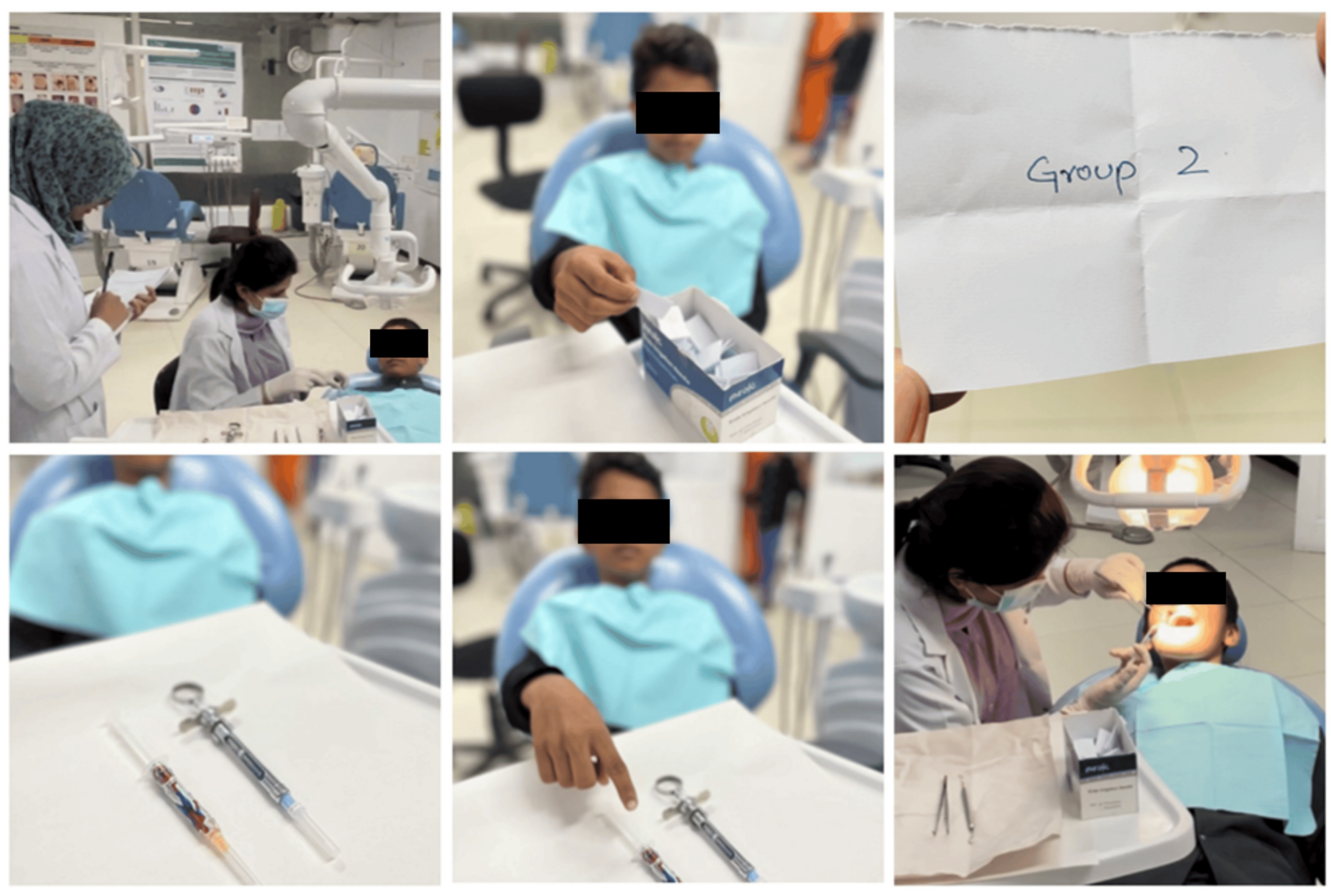
Illustration of key steps in selection of the injector by the child

The “Tell, Show, Do” technique was used to explain the procedure to the child and their parents. Topical anesthesia was applied to the injection site. The injector was loaded with LA. The dentist then administered the LA using a child’s preferred injector, while an observer, positioned discreetly behind the child, documented the child’s behavior using Frankl behavior rating scale. This rating ranges from score 1 to 4, with rating 1 being definitely negative and rating 4 being definitely positive. Figure 2 illustrates the stepwise process described above.

Data entry and statistical analysis were performed using SPSS version 25 (IBM Corp., Armonk, NY). Quantitative variables were expressed as mean and standard deviation, while qualitative variables were presented as frequency and percentages. The association between study groups and Frankl behavior rating scales were evaluated using the Chi-square test. A p-value of ≤0.05 was considered statistically significant.

## Results

The demographic details of the study are outlined in Table 1. Group A had a mean age of 8.13 ± 2.33 years, while Group B had a slightly younger mean age of 7.27 ± 2.16. The age range for both groups spanned from 4 to 12 years.

**Table 1:** Comparison of preferences for metal vs. plastic injectors (with and without stickers) between Group A and Group B

	Group A		Group B	
	n	%	n	%
Metal injection	32	42.7%	13	17.3%
Plastic injection without stickers	43	57.3%	-	-
Plastic injection with stickers	-	-	62	82.7%

Regarding the preference for dental injectors, in Group A, 42.7% (n = 32) of the children selected the metal injectors, while 57.3% (n = 43) opted for plastic injectors without stickers. In Group B, a significant 82.7% (n = 62) of the children preferred the plastic injector with stickers, compared to only 17.3% (n = 13) who chose the metal injector. The majority of the children demonstrated a clear preference for plastic injectors, especially those with stickers (Table 1).

When asked about their reasons for choosing a particular injector, children in Group A who selected the plastic injector often did so due to its less intimidating nature (74.4%, n = 32), while 34.3% (n = 11) chose the metal injector out of intrigue, perceiving it as unfamiliar or interesting. Additionally, 74.4% (n = 32) found the plastic injector less intimidating than the metal option. However, in Group B, 75.8% (n = 47) of children were attracted to the plastic injector because of its sticker, which made it visually appealing, while 38.4% (n = 5) chose metal injectors, finding it intriguing. They thought it was a device or a cool gadget rather than an injector. These results highlight that both appearance and familiarity significantly influenced children’s preferences, with the stickers on the plastic injectors making them more appealing to the majority of children (Table 2).

**Table 2:** Children's fear, comfort, and preference responses to metal vs. plastic injectors (with and without stickers) across Group A and Group B

Group	Group A				Group B			
Options	Metal injection, n = 32		Plastic injection without stickers, n = 43		Metal injection, n = 13		Plastic injection with stickers, n = 62	
	n	%	n	%	n	%	n	%
Fearful of metal injector	0	0%	4	9.3%	0	0%	5	8.1%
Comfortable with metal injector	2	6.3%	0	0%	0	0%	0	0%
Uncomfortable with plastic injectors	12	37.5%	0	0%	4	30.8%	0	0%
Plastic injectors seem less painful	0	0%	7	16.3%	0	0%	3	4.8%
Metal injectors seem less painful	3	9.4%	0	0%	0	0%	0	0%
Plastic injector appears less intimidating	0	0%	32	74.4%	0	0%	4	6.5%
Metal injectors appear less intimidating	1	3.1%	0	0%	0	0%	0	0%
Prefers metal injector	3	9.4%	0	0%	4	30.8%	0	0%
Intrigued by stickers on the plastic injector	0	0%	0	0%	0	0%	47	75.8%
Intrigued by unfamiliar metal syringe	11	34.3%	0	0%	5	38.4%	3	4.8%

When analyzing children’s behavior using the Frankl behavior rating scale, combining the positive and definitely positive responses together to one entity (positive behavior), the results reveal a clear preference for plastic injectors. In Group A, 67.5% (n = 29) of the children who chose the plastic injector without stickers showed a positive response compared to only 31.3% (n = 10) of those who selected metal injectors with a statistically significant p-value. In Group B, 69.3% (n = 43) of children who opted for plastic injectors with stickers displayed positive behavior, while 46.2% (n = 6) of those who chose the metal injector did the same. Negative behaviors were more prevalent among children using metal injectors in both groups. These results strongly favor that the appearance of the injectors, particularly when enhanced with child-friendly stickers, plays a crucial role in improving children’s comfort and cooperation during dental procedures (Table 3).

**Table 3:** Frankl behavior score comparison between metal and plastic injectors (with and without stickers) across Group A and Group B *Significant p-value.

Frankl behavior score		Group A				Group B			
		Metal injection, n = 32		Plastic injection without stickers, n = 43		Metal injection, n = 13		Plastic injection with stickers, n = 62	
		n	%	n	%	n	%	n	%
Definitely negative	1	13	40.6%	8	18.6%	6	46.2%	10	16.1%
Negative	2	9	28.1%	6	14%	1	7.7%	9	14.5%
Positive	3	4	12.5%	22	51.2%	5	38.5%	24	38.7%
Definitely positive	4	6	18.8%	7	16.3%	1	7.7%	19	30.6%
P-value	0.005*					0.071			
Chi-square values	12.995					7.040			

Across all the age groups, the Frankl behavior rating results, combining “positive” and “definitely positive” as positive behavior, consistently showed that children responded more favorably to plastic injectors compared to metal ones, especially when the plastic injectors were covered with stickers. In the youngest age group (4-6 years), 73.3% (n = 22) of children responded with positive behavior to plastic injectors with stickers, compared to only 33.3% (n = 7) for metal injectors, which was statistically significant (p = 0.039). Similarly, in the 7-10 years age group, plastic injectors - both with and without stickers - elicited positive responses in 68% (n = 17) and 75% (n = 15) of children, respectively, which was also statistically significant (p = 0.011). Even among the oldest children (11-12 years), plastic injectors without stickers led to the highest positive response rate (83.4%, n = 10), though the difference was not statistically significant. Overall, the data suggest that plastic injectors, particularly those with stickers, are more effective in promoting positive behavior and reducing anxiety during LA injections and the following dental procedures (Table 4).

**Table 4:** Age-wise comparison of children's responses to metal and plastic injectors (with and without stickers) *Significant p-value.

Age groups	Group	Definitely negative	Negative	Positive	Definitely positive	p-value	Chi-square values
4-6 years, n = 62	Metal injection	10	4	4	3	0.039*	13.456
		47.6%	19.0%	19.0%	14.3%		
	Plastic injectors without stickers	4	3	3	1		
		36.4%	27.3%	27.3%	9.1%		
	Plastic injectors with stickers	5	3	13	9		
		16.7%	10.0%	43.3%	30.0%		
7-10 years, n = 64	Metal injection	7	6	4	2	0.011*	16.556
		36.8%	31.6%	21.1%	10.5%		
	Plastic injectors without stickers	4	1	11	4		
		20.0%	5.0%	55.0%	20.0%		
	Plastic injectors with stickers	3	5	8	9		
		12.0%	20.0%	32.0%	36.0%		
11-12 years, n = 24	Metal injection	2	0	1	2	0.253	7.802
		40.0%	0.0%	20.0%	40.0%		
	Plastic injectors without stickers	0	2	8	2		
		0.0%	16.7%	66.7%	16.7%		
	Plastic injectors with stickers	2	1	3	1		
		28.6%	14.3%	42.9%	14.3%		

## Discussion

The two groups, one with stickers and one without, were formed to determine if the positive behavior was due to the stickers or the plastic injectors themselves. The selected age group, 4 to 12 years, is crucial for psychological and emotional development, particularly in relation to dental anxiety and fear of injectors [19]. Children in this age group are sensitive to new experiences, making dental treatment potentially stressful to them. The results showed a strong preference for plastic injectors (particularly with stickers) over traditional metal injectors. In Group B, 82% of children chose plastic injectors with stickers, suggesting the visual appeal of these injections played a significant role in their decision, thus improving cooperation during dental procedures. A possible explanation for this could be that children might already be familiar with plastic injectors due to prior medical treatments.

Familiarity and visual appeal can reduce anxiety, particularly in younger children, and this plays a crucial role in their selection. During this developmental stage, children's emotional responses and coping mechanisms are significantly shaped by their social interactions with their peers and caregivers. Additionally, children in this age group frequently visit dental clinics for routine checkups and dental issues, making it an ideal time to study and address dental anxiety [20]. Assessment of anxiety development during this period allows for early intervention, potentially preventing long-term DFA.

The Frankl behavior rating scale was chosen to assess children’s behavior during LA. This scale was chosen because preschool-aged children often struggle to verbally express their emotions accurately, making behavioral observations a more reliable method for assessing anxiety and cooperation [21]. Our findings indicated a strong preference for plastic injectors, especially those with stickers, among young children, and this was correlated with more positive behavior during treatments. Saleem also divided their participants into two groups and found similar results where children preferred plastic injectors with stickers (78.39%) when given the option, as opposed to those without stickers (34.5%) [18]. This is also consistent with a study conducted by Kohli et al., who found that using insulin and deception syringes significantly reduced children's anxiety as evidenced by lower postoperative anxiety score and reduced pain perception using Venham’s picture scale and Wong Baker face pain rating scale, respectively [8].

Similarly, Melwani et al. documented reduced DFA and lower pulse rates in children using camouflaged syringes, although the difference was not statistically significant [10]. These findings support the notion that modifications to the visual and tactile aspects of dental injectors can positively influence children’s experiences during their dental visit.

However, it is important to note that the influence of such modification may diminish with age, as seen in our study. Older children, with more advanced cognitive abilities (11-12 years of age), showed less interest in injectors with stickers. This aligns with the findings from Shao et al., who suggested older children may perceive visual modification like stickers as childish and be more focused on the function of the injector itself [22].

Ludovichetti et al. demonstrated that computerized anesthesia delivery systems reduce pain perception and promote cooperative behavior in young patients with statistically significant differences among electronic versus traditional anesthesia [23]. Though the approaches differ, these findings support our study’s conclusion that innovative anesthesia technique improves pediatric dental experience. Our study found that camouflaged injectors with stickers led to a more pronounced positive behavior, particularly among young children. Versloot et al., however, found no significant difference in pain or distress between the Wand computerized anesthesia system and traditional systems, noting that highly anxious children exhibited similar distress regardless of the method, suggesting baseline anxiety plays a critical role in anesthesia administration [24].

In contrast, some studies have found that behavioral interactions during these dental procedures may not always be effective. Kharouba et al. observed that handholding during injections did not significantly impact pain, anxiety, and cooperation [25]. Similarly, Thoppe-Dhamodhara et al. found no significant difference in children’s behavior when comparing conventional and computer-controlled anesthesia delivery systems (CCADS) [26]. This divergence underlines a variety of factors that can influence children’s responses to dental procedures and suggests that visual and tactile modifications to injectors may be more effective in some contexts than others.

Distraction techniques like audiovisual (AV) methods and immersive virtual reality have proven effective in managing DFA and enhancing cooperation in pediatric dentistry. Patil et al. demonstrated that AV distractions significantly lowered anxiety levels in children aged 6-10 during LA administration with statistically significant results on VAS and Houpt scale [27]. Similarly, Muhammad et al. found AV distractions significantly lowered anxiety levels in children during dental treatment using FIS and modified Venham scale [28]. Ijaz et al. emphasized the importance of visual appeal and innovation in improving children’s comfort and cooperation during these dental procedures [29]. These findings suggest introducing child-friendly injector designs alongside a child-friendly clinic environment to create a more soothing experience for children. Furthermore, providing children with choices regarding their treatment may help reduce their DFA.

When children were asked why they chose a specific injector type, responses varied in terms of familiarity and visual appeal. In Group A, more than one-third (36.3%) of children preferred plastic injectors, citing familiarity from past medical and dental experiences. Meanwhile, 34.4% found metal injectors intriguing due to their unfamiliarity, viewing them as a novel tool rather than a standard syringe. In Group B, the addition of stickers to the plastic injectors played a significant role, with 75.8% selecting them as they appear friendlier and less intimidating. Interestingly, 41.7% of children in the same group were drawn to metal injectors due to their unfamiliarity, making them interesting. Similar findings were reported by Saleem, where children perceived the plastic injector as less reliable, with some expressing concerns that they might break during the procedure [18].

Despite offering valuable insights, this study has several limitations. The relatively small sample size limits generalizability and precision of the findings. A larger sample would improve the reliability of the results. Additionally, the focus on specific geographic and socioeconomic demographics may not fully represent children from diverse backgrounds. Moreover, this study does not account for other potentially influential factors such as prior dental experiences, individual psychological profiles, or the presence of parental support during procedures. Future research should address these limitations by including a larger and more diverse multicenter study to enhance the generalizability and better address DFA in children.

## Conclusions

The study highlights how the appearance of dental injectors can meaningfully impact children’s behavior during dental procedures. Younger children responded more positively and showed better cooperation when plastic injectors, especially those decorated with stickers, were used. In contrast, older children were less influenced by the injector’s appearance, suggesting that their preferences and perceptions evolve with age. These findings underline the importance of considering children’s comfort and emotional needs - tailored by age - when planning pediatric dental care.
